# Effects of Altitude on Tea Composition: Dual Regulation by Soil Physicochemical Properties and Microbial Communities

**DOI:** 10.3390/plants14111642

**Published:** 2025-05-28

**Authors:** Xirong Ren, Minyao Lin, Jiani Liu, Waqar Khan, Hongbo Zhao, Binmei Sun, Shaoqun Liu, Peng Zheng

**Affiliations:** College of Horticulture, South China Agricultural University, Guangzhou 510642, China; xirong_tea@163.com (X.R.);

**Keywords:** tea quality, soil chemical properties, rhizosphere soil microbial communities, altitudinal gradient

## Abstract

Soil chemical properties and soil microbial communities are the key factors affecting the content of tea. The mechanism by which altitude changes soil’s chemical properties and microbial community structure to affect tea content is unclear. This study was conducted on a typical tea plantation in the Fenghuang Mountains of Chaozhou, China. It systematically revealed the relationship between soil chemical properties and microbial communities with tea quality components between different altitudes (396 m/517 m/623 m). We discovered that soil pH and soil Catalase activity appeared to decrease and then increase with altitude, and soil SOM content and soil Acid Phosphatase activity were significantly higher at mid-altitude. Soil TP and TK content were lowest at high altitudes (0.20 mg/kg, 5.98 mg/kg). Non-significant differences were found in the spatial composition of microbial communities at different altitudes. The abundance of fungi (Sobol index) was significantly higher (*p* < 0.05) at low altitudes than in other altitude groups. Redundancy analysis indicated that soil pH and TP are drivers of changes in bacterial community structure. The abundance of Fibrobacteres, a key functional group of bacteria, showed a decreasing trend with increasing altitude, and *Stachybotrys* (fungi) likewise had the lowest abundance at high altitude (*p* < 0.05). The catechin, theanine, and caffeine content of tea leaves accumulated the least at high altitude (12.91%, 0.39%, 2.88%). Fibrobacteres and *Stachybotrys*, as well as soil TK and TP content, were strongly associated with the accumulation of major contents in tea leaves. Meanwhile, fungal abundance was significantly and positively correlated with theanine (*p* < 0.05). This study enhances our understanding of soil chemical property–soil microbial community–tea tree interactions. By exploring the differences in soil key nutrient content and the abundance of functional flora driving tea quality at different altitudes, it provides a basis for the precise microecological management of tea gardens.

## 1. Introduction

The tea plant [*Camellia sinensis* (L.) O. Kuntze] is one of the most important economic crops in China. Tea cultivation is concentrated in the regions of China, India, Kenya, and Sri Lanka. China is the largest grower and exporter of tea and has a long history of drinking it. The flavor of tea is mainly derived from the secondary metabolites of tea, and catechins, theanine, and caffeine are regarded as key indicators affecting the quality of tea. The strength and mellowness of tea flavor lies in the high theanine content and moderate catechin content in tea. The high content of theanine and caffeine is the reason for the bitterness and astringency of tea [1,2]. Catechins mainly contain ester catechins and non-ester catechins, and the ester catechins mainly contain EGCG and ECG, which are the factors causing the strong astringent flavor of tea. Non-ester catechins, mainly EGC, are weakly astringent, have a strong sweet aftertaste, and have an important synergistic effect on the flavor of tea broth. The balance of catechins, theanine, and caffeine contributes to the overall flavor and unique characteristics of tea [3].

The secondary metabolites in tea are regulated in multiple ways by the environment in which the tea plant grows. Altitude differences lead to changes in temperature, sunlight intensity, and soil chemical properties for tea tree growth. The catechin content of tea usually decreases with increasing altitude, and the theanine content is positively correlated with altitude. The increase in the theanine-to-catechin ratio in high mountain environments [4] is believed to be a significant reason for the higher quality of tea at high altitude. Changes in elevation lead to changes in many environmental factors, which in turn lead to changes in the diversity and composition of microbial communities in the soil [5]. Nutrient substances, microbial composition, and the structure of the soil influence the accumulation of secondary metabolites in tea leaves [6,7]. The extensive current research focuses on the improvement of soil properties and enhancement of microbial community diversity in the soil through fertilization and intercropping in tea gardens. This affects the amino acid and flavonoid metabolism of tea plants and enhances tea quality. Huang et al. revealed that the key effects of soil pH and *Betaproteobacteria* abundance crucially affect nitrogen transformation [8]. Likewise, Shao et al. found that intercropping increased the abundance of microbial taxa involved in organic matter decomposition, such as *Pseudomonas*, *Sphingomonas*, and *Archaeorhizomyces*, which in turn enhanced tea growth and yield [9]. The composition of soil microbial communities in tea plantations also varies according to the type of soil. Differences in soil types alter microbial community diversity and ecological functions, which regulate the formation of different qualities of tea [10]. The effects of soil nutrients and microorganisms in tea plantations on the growth and metabolism of tea tree have been well studied. However, there are still insufficient studies on the relationship between changes in the soil microbial environment driven by the altitudinal gradient and secondary metabolites of tea tree.

Nutrient composition and microbial communities in the soil influence tea plant metabolism [7]. Soil organic matter (SOM), nitrogen (N), phosphorus (P), potassium (K), pH, and soil enzyme activity are essential indicators of soil fertility [11,12]. One study showed that nutrient deficiencies in the soil decreased theanine in tea, increased the phenol–ammonia ratio in tea, and decreased tea quality [13]. Soil quality is simultaneously regulated by the soil’s microbial community. Soil microorganisms are driving forces in soil ecosystems responsible for nutrient cycling, organic matter decomposition, pest and disease control, and plant growth and development [14,15]. The microbial community in the soil also affects the quality of tea. Microbial communities in the root system of the tea tree, such as Ascomycetes and Actinomycetes, can increase ammonia uptake through nitrogen metabolism in the soil and promote the synthesis of theanine in tea, and the accumulation of polyphenols is promoted by Bacillus in the soil [16]. Elevation changes lead to differences in the microclimate in which the tea plant grows, including changes in the temperature and humidity in which the tea plant grows. Soil chemical properties and microbial communities are susceptible to environmental changes [17]. A study found that soil bacterial and fungal community diversity and abundance increased with an increasing altitude gradient, and significant differences in microbial community structure were observed between low and high altitudes [18]. Soil chemical properties are at the same time key factors influencing the diversity and composition of soil microorganisms, and previous studies have found [19,20] that the differences in altitude lead to changes in the spatial environment for tea plant growth. Therefore, it is essential to understand the interrelationships between soil chemical properties and soil microbial communities in tea plantation soils in different spaces, as well as to further study the relationship between soil microbial communities and tea quality components.

Tea plant–soil chemical properties and microbial community structure interactions exist and are important for sustainable tea production [15]. In this study, we analyzed the diversity and composition of soil microorganisms at different altitudes using high-throughput sequencing, and explored the relationship between tea tree rhizosphere microorganisms, soil chemical properties, and tea quality components. Linking tea quality to soil provides new insights into the relationship between soil microbial communities in the tea tree rhizosphere and tea quality, and facilitates the development of efficient soil management strategies to improve tea quality.

## 2. Results

### 2.1. Rhizosphere Soil Properties and Root Traits Varied with Altitudes

**Figure 1:**
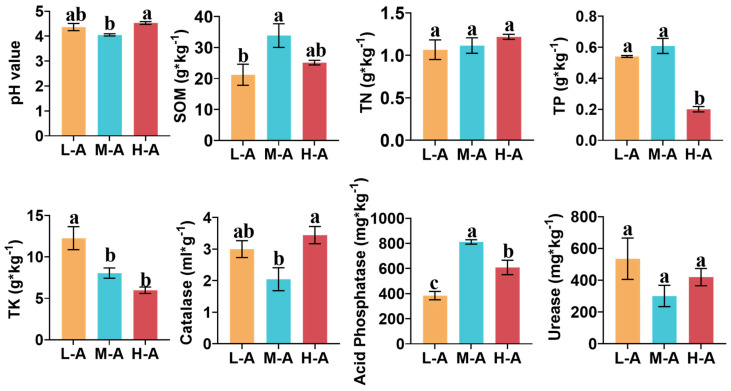
The soil chemical properties of tea farms at three different altitudes. L-A, low altitude, M-A, mid-altitude, H-A, high altitude. Soil chemical properties included soil pH, soil organic matter content (SOM), total nitrogen (TN), total phosphorus (TP), and total potassium (TK). Soil enzyme activities include Catalase, Acid Phosphatase, and Urease. The data represent means ±standard error of the mean (SEM) (n = 3); Different lowercase letters indicate significant differences among groups (*p* < 0.05, Tukey’s HSD post hoc test).

The soil chemical properties of tea tree root systems at different altitudes were significantly different, as shown in Figure 1. Soil pH decreased by 7.3% and 10.5% in the mid-altitude (M-A) compared to the low altitude (L-A) and high altitude (H-A), respectively. Soil Catalase enzyme activity followed the same trend as soil pH at different altitudes, with a significant decrease in enzyme activity at mid-altitude (M-A). There were differences in soil nutrient content at different elevations. Soil organic matter content (SOM) was the highest at mid-altitude (M-A) and increased by 59.5% over that at high altitude (H-A). Soil Acid Phosphatase enzyme activity and SOM showed the same trend at different altitudes, with Acid Phosphatase enzyme activity at mid-altitude (M-A) being 111.3% and 33.5% higher than that at low altitude and high altitude, respectively. With altitude, SOM and Acid Phosphatase enzyme activity peaked at mid-altitude (M-A). Soil total phosphorus (TP) content was significantly at its lowest at high altitude (H-A), and rooted soil total potassium (TK) content showed a decreasing trend with increasing medium altitude. Information on soil chemical properties is provided in Table S1.

### 2.2. Microbial Community Compositions and Diversity

**Figure 2:**
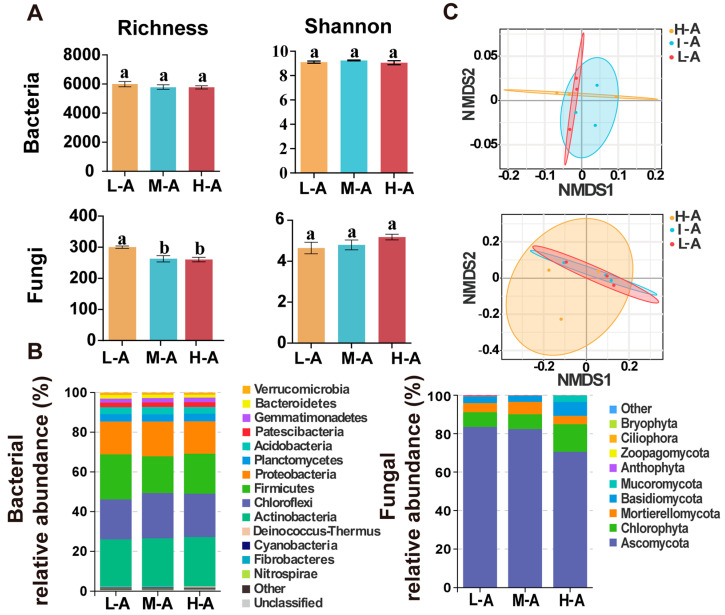
(**A**) Richness and Shannon index of soil bacterial and fungal alpha-diversity. Different letters over the plots indicate significant differences (*p* < 0.05). (**B**) The relative abundance of the dominant bacterial and fungal phyla from the soil. (**C**) The relative abundance at the phylum level and the non-metric multidimensional scaling (NMDS) of microbial communities. L-A, low altitude, M-A, mid-altitude, H-A, high altitude.

Bacterial and fungal diversity were observed using the rhizosphere soil microbial community richness (Sobol index) and the Shannon index (Figure 2A). Soil bacterial richness (Sobol index) and diversity did not change significantly at different elevations. The abundance of fungal communities in the soil at different altitudes differed significantly, with the abundance of fungi at low altitude (L-A) being significantly higher than that of the other altitude groups (*p* < 0.05) (Figure 2A). At the phylum level, the dominant soil bacterial populations at different elevations were mainly Actinobacteria (23–24%), Chloroflexi (20–23%), Firmicutes (18–23%), and Proteobacteria (16–23%), and these dominant populations together accounted for about 90% of the total bacterial abundance above. The relative abundance of bacterial Fibrobacteres was significantly higher at low altitude (L-A) than in the other altitude groups (*p* < 0.05). Ascomycota (70–83%), Chlorophyta (7–14%), Basidiomycota (3–7%), and Mortierellomycota (4–6%) were the dominant fungal phyla of the soil at all elevations (Figure 2B). The composition of the dominant bacterial and fungal communities in the rhizosphere soil of tea tree did not show significant differences at the phylum level at different altitudes (*p* > 0.05). The results indicated that the dominant microbial populations in the soil are stable over an altitudinal gradient. At the fungal genus level, *Stachybotrys* (Ascomycota) showed significant spatial variation in elevation, with the relative abundance of the mycorrhizal flora being significantly higher at low altitude (L-A) and mid-altitude (M-A) than at high altitude (H-A) (Figure S1). In the NMDS results map (Figure 2C), it was found that intra-group heterogeneity was high, and the microbial community structure was highly variable within the same altitude. Differences in the spatial composition of microbial communities at different altitudes were not significant.

### 2.3. Relationship Between Soil Chemical Properties and Microbial Relative Abundance

**Figure 3:**
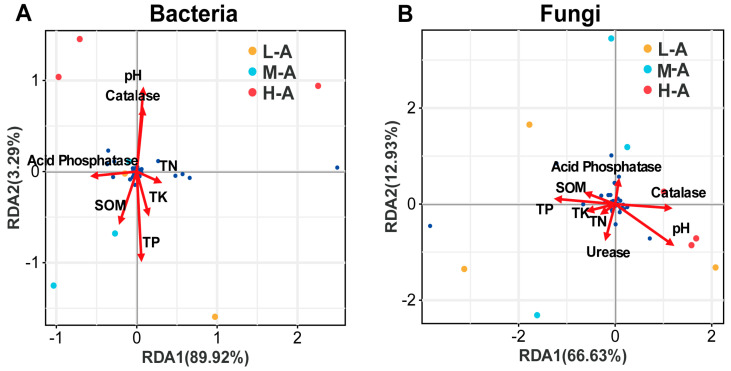
(**A**) Result of redundancy analysis of rhizosphere soil bacterial communities (at genus level) in relation to soil properties and root traits. (**B**) Result of redundancy analysis of rhizosphere soil fungal communities (at genus level) in relation to soil properties and root traits. Ordination diagrams present species scores (blue) and environmental factor scores (red lines) in redundancy analysis. Soil chemical properties include soil pH, soil organic matter content (SOM), total nitrogen (TN), total phosphorus (TP), and total potassium (TK). Soil enzyme activities include Catalase, Acid Phosphatase, and Urease.

To determine the effect of soil chemical properties on the soil microbial community composition in the root system of tea trees, RDA was used. In the bacterial community, the first two constraint axes explained 93.21% of the total (Figure 3A). It was found that soil pH (*R*^2^ = 0.79, *p* = 0.011) and soil TP (*R*^2^ = 0.90, *p* = 0.003) significantly influenced the soil bacterial community structure. In the fungal community, the first two constraint axes explained 79.23% (Figure 3B), while soil chemical properties had no significant effect on soil fungal community structure (*p* > 0.05).

We conducted a correlation analysis of soil chemical properties with soil microbial communities (top 20 species in abundance at phylum and genus level) (Table 1). The results revealed a strong correlation between soil microbial communities, soil chemical properties, and soil enzyme activities. In the bacterial community, Fibrobacteres were positively correlated with TP and TK in soil and significantly negatively correlated with soil pH (*p* < 0.05). The fungal community *Stachybotrys* (Ascomycota) was negatively correlated with soil pH and Catalase and strongly positively correlated with TP (*p* < 0.05).

### 2.4. Relationship of Soil Chemical Properties and Microbial Communities with Tea Quality

**Figure 4:**
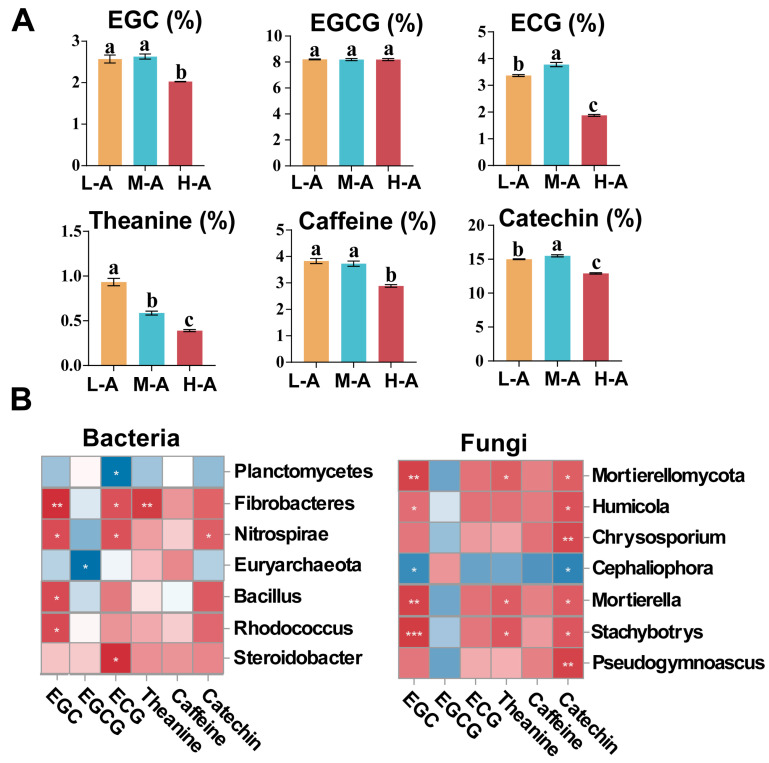
(**A**) The major composition of tea leaves. The data represent means ± standard error (n = 3). Letters denote statistically significant differences. EGC, Epigallocatechin; ECG, Epicatechin-3-gallate; EGCG, Epigallocatechi gallate; Catechin, total catechin content. (**B**) Correlation analysis between the major composition or quality indices of green tea and the relative abundance of a dominant phylum or genus. Asterisks indicate statistical significance (* *p* < 0.05, ** *p* < 0.01, and *** *p* < 0.001).

The major components of tea at different altitudes were significantly different from each other (Figure 4A). With increasing altitude, the content of theanine in tea leaves gradually decreased (*p* < 0.05). The lowest content was reached at high altitude (H-A), while the caffeine content in tea was also lowest at high altitude (*p* < 0.05). The theanine content was reduced by 58.2% and 33.9% and the caffeine content was reduced by 24.8% and 22.5% at high altitude (H-A) compared to low altitude (L-A) and mid-altitude (M-A), respectively. The total catechins in tea were significantly higher (*p* < 0.05) at the mid-altitude (M-A) than at other altitudes, mainly due to the fact that the EGC and ECG contents of catechins were the highest at the mid-altitude (M-A). There was no significant change in the EGCG content of catechins at different altitudes.

We conducted correlation analysis of the soil chemical properties with major tea leaf components (Table S2). Theanine in tea was found to be significantly positively correlated with TK in soil (*p* < 0.05). Caffeine and catechin were significantly correlated with TP in soil (*p* < 0.05), and pH and Catalase in soil were negatively correlated with catechin (*p* < 0.05). The theanine/caffeine ratio is a measure of the sweetness and bitterness of tea. The theanine/catechin ratio is a measure of the sweetness and astringency of tea, and higher values of the two ratios correspond to a higher quality of tea [21]. In the quality analysis, a strong positive correlation was found between TK in soil and tea quality (*p* < 0.05), while Acid Phosphatase in soil showed a strong negative correlation with tea quality (*p* < 0.05).

Analysis of the correlation between soil microbial abundance and tea leaves’ main internal components was conducted (Table 2). There was a strong correlation between soil fungal abundance and tea quality components, with a significant positive correlation mainly with theanine content in tea leaves (*p* < 0.05). Theanine/caffeine and Theanine/catechin were both significantly and positively correlated with the abundance of fungi in the soil (*p* < 0.05). Meanwhile, there was a strong positive correlation between TK and the abundance of fungal communities in the soil (Table S3).

The correlation of major biochemical components in tea with soil microbial communities (top 20 species in terms of abundance at phylum and genus level) was conducted (Figure 4B). The results revealed a strong correlation between the soil microbial community and the biochemical composition of tea. The results showed that Fibrobacteres in the bacterial community were positively correlated with EGC, ECG, and theanine in tea leaves (*p* < 0.05). The fungal community of *Stachybotrys* (Ascomycota) showed strong positive correlations with total catechins, EGC, and theanine (*p* < 0.05).

## 3. Discussion

### 3.1. Soil Chemical Properties at Different Altitudes

With the increase in altitude (Table 3), the average temperature of the tea hills appeared to be significantly lower and the precipitation appeared to be higher. Changes in the growing environment of tea trees have led to differences in soil chemical properties [22]. Soil pH is critical to the growth and development of tea trees, and altitude factors are essential factors influencing soil pH changes [23]. In this study, soil pH appeared to decrease (Figure 1) and then increase with increasing altitude. The trend of soil pH change from low altitude to mid-altitude is consistent with previous studies [24,25]. However, at high altitude, soil pH again showed an increasing trend. We hypothesized that it might have been related to the decrease in ambient air temperature with increasing altitude, which reduced the evaporation of soil moisture and allowed for the accumulation of alkaline substances [26]. As altitude changed, the highest SOM occurred in soils at mid-altitude (Figure 1). Low temperature conditions at high altitude promoted the accumulation of C in the soil, while higher temperature conditions at low altitude promoted the accumulation of polysaccharides and other substances and reduced soil C accumulation. Therefore, at mid-altitude, due to the suitable temperature, the soil had a high SOM content [27]. TP and TK were enriched at low altitude (Figure 1). This was probably due to the relatively gentle topography and slow water flow rate at low altitude meaning that less TP and TK were carried away by leaching, and more of them were accumulated in the soil [28,29]. Soil enzyme activity is an effective driver of organic matter decomposition and nutrient transformation in soils and has a key role in improving soil quality [30]. In our study, it was interesting that Catalase activity decreased with increasing altitude, whereas Acid Phosphatase appeared to increase and then decrease in activity. High soil Acid Phosphatase activity at mid-altitude may have been affected by the high SOM in the soil, accelerating TP release from the soil [31] and thereby maintaining higher soil nutrients. Since Catalase activity depends on soil pH and increases with improved soil acidic properties, Acid Phosphatase activity in the soil significantly increases at high altitude. Elevation difference affects soil chemical properties and enzyme activity in tea plantation soil, which can reveal the rule of changes in vertical space in tea plantations caused by elevation differences.

### 3.2. Soil Microbial Composition at Different Altitudes

According to the NMDS results, the spatial composition of the soil microbial community was found to be insignificantly different at different altitudes. Changes in altitude combined with changes in various environmental factors, soil temperature, and humidity as the altitude gradient increased. The altitude gradient changed environmental factors such as soil pH, TP, soil temperature, and precipitation by altering soil pH, TP, soil temperature, and precipitation. The indirect effect of environmental factors may have masked the direct effect of altitude on microbial communities [32]. The structure of soil bacterial and fungal dominant communities was similar at different elevations. The composition of soil-dominant communities in tea plantations was consistent with previous studies [6]; the soil dominant bacteria (phylum) were Actinobacteria, Chloroflexi, and Firmicutes, and the dominant fungi (phylum) were Ascomycota and Chlorophyta (Figure 2). Variations in fungal richness are usually significantly correlated with temperature, with changes in temperature induced by differences in altitude. This leads to significant differences in the abundance of fungal communities, whereas bacteria are less sensitive to altitude than fungi [33]. In previous studies, the abundance of soil fungal communities was significantly and positively correlated with SOM [34,35], whereas in the present study, an increase in fungal community diversity was found to occur in soil conditions with low SOM (*p* < 0.05). It is hypothesized that because of the high environmental stress in low-nutrient soils, multiple species of fungi have evolved different survival strategies and ecological functions to adapt to the soil environment, thus contributing to the increase in fungal diversity [36]. Thus, soil fungal community richness was highest at low altitude under the synergistic effect of environmental factors and nutrient limitation-driven ecotone differentiation strategies. Meanwhile, a significant positive correlation was found between soil TK content and soil fungal diversity (Table S3), suggesting that soil K content is crucial in explaining the variation in fungal abundance with altitude [37]. Intense competition exists in the soil community structure, and it has been found in previous studies that higher nutrient levels in the soil usually appear to be accompanied by a low diversity of microorganisms in the soil [18,38]. The highest abundance of Fibrobacteres in the bacterial community at low altitude was explained by the low soil organic matter content at low altitude (Figure 2). The fungal genera *Stachybotrys* (Ascomycota) play a role in the soil by decomposing organic matter and promoting the cycling of carbon, nitrogen, and phosphorus in the soil [39]. We found that soil pH and TP content significantly affected the relative abundance of *Stachybotrys* (Ascomycota) depending on altitude (Table 1) and that acidic soils were more suitable for the growth and reproduction of this bacterial group [40], explaining the decrease in relative abundance at high altitudes.

### 3.3. Key Drivers of Change in Soil Fungal and Bacterial Communities

In the NMDS analysis, it was found that the differences in communities at different altitudes were not significant. However, RDA pointed out that pH and TP are key factors for bacterial communities. This could mean that altitude itself is not a direct influence but works indirectly by affecting pH and TP in the soil. Under acidified soil conditions, the activity of bacterial communities is limited. Soil bacteria have a narrow range of pH suitable for growth, whereas fungi can maintain a stable structure over a wide range of soil pH. Therefore, soil pH is also influenced by soil TP content [41]; soil TP drives accelerated soil acidification, and soil acidification inhibits the growth of other competing microbial taxa [42]. Therefore, soil pH and TP are core drivers of bacterial communities (Figure 3). Bacteria Fibrobacteres are a group of bacteria whose primary function is cellulose degradation [43]. The high level of TP and TK may indirectly support the metabolism of Fibrobacteres by activating phosphorus-dependent cellulases [44]. At the same time, low Catalase activity leads to H_2_O_2_ accumulation, which may inhibit some microbial growth in the process, whereas high TP directly supports the phosphorus metabolic requirements of *Stachybotrys*. It may simultaneously inhibit the competition of some bacteria and further enhance their ecological niche advantage [45]. Therefore, at low altitude, the synergistic effect of TK and TP enrichment and the slightly acidic environment together drove a significant increase in the relative abundance of Fibrobacteres and *Stachybotrys* in the soil. We found, interestingly, that although soil chemical properties and soil enzyme activities did not significantly affect the composition of the soil fungal community, there was a strong correlation with some of the soil species (Table 1). pH is mostly negatively correlated with species at the phylum and genus level of the fungal community in the soil [46]. It is assumed that soil pH affects the decomposition of organic matter in the soil, which indirectly affects the nutrient sources of fungi [47], resulting in a decrease in the number of some organic nutrient-dependent fungal genera. The TP in soil can provide more phosphorus sources for fungi, promote energy metabolism, and support fungal growth and reproduction [48]. Therefore, TP showed a strong positive correlation with species at the phylum and genus levels of fungi.

### 3.4. Relationship Between Soil Chemical Properties and Tea Quality

There was a significant effect of variation in the major internal components of tea at different altitudes (Figure 4). Catechins are the main source of astringency in tea broth [49]. The lowest accumulation of catechin content being in tea at high altitude is consistent with the results of previous studies [50]. At higher altitudes, lower temperatures and light scattering inhibit catechin synthesis [51]. Diffuse light at high altitude facilitates the nitrogen metabolism of the tea tree to produce more amino acids and nitrogenous compounds [51,52]. However, our study found that the theanine content decreased with increasing altitude. Soil TP and TK can provide phosphorus and potassium nutrients for tea trees, which is favorable to the accumulation of catechins in the new green shoots of tea trees [53]. In our study, we found a strong positive correlation between theanine soil TK content, so we hypothesized that the synthesis of theanine might be related to soil TK content. Soil K has been shown in previous studies to ameliorate soil drought stress while inducing the production of more free amino acids by the tea tree and also boosting tea’s caffeine content [54,55]. Soil P has been reported to promote photosynthesis in tea trees to enhance the sugar content in tea leaves, which is converted to polyphenols, resulting in the increased accumulation of catechin content in tea leaves [56]. In this study, catechin accumulation in tea leaves was affected by both the growing environment of tea trees at high altitude and the lower TP content. The higher the theanine/catechin and theanine/caffeine ratios, the higher the quality and economic value of tea leaves. In the present study, it was found that the ratio of two key indicators characterizing the quality of tea broth was highest at low altitude. Therefore, we hypothesized that in this study, it was mainly regulated by soil chemical properties rather than the altitude factor. Therefore, the high TK content in the soil at low altitude is more beneficial to the accumulation of secondary metabolism in the tea leaves and enhances the flavor of the tea broth. Meanwhile, in this study, we found a strong negative correlation between soil Acid Phosphatase activity and tea quality. Elevated Acid Phosphatase activity reflects the lack of effective phosphorus in the soil. When the soil is deficient in phosphorus, the tea tree may use more resources for the acquisition of P elements, leading to the restriction of nitrogen metabolic pathways [57], so phosphorus limitation may affect the quality of tea by indirectly affecting the efficiency of nitrogen metabolism and reducing the synthesis of theanine.

### 3.5. Relationship Between Soil Microbial Community and Tea Quality

The composition and structure of the rhizosphere soil microbial community of the tea tree are critical for the growth and development of the tea tree [58]. We analyzed the diversity of the microbial community in a tea plantation and the quality-related components of the tea leaves, which were the same as the results of study [59]. Fungal diversity showed a significant positive correlation with theanine (Table 2) and a robust positive correlation with theanine/caffeine and theanine/catechin. The results showed that at low altitude, the diversity of rhizosphere soil fungi enhanced ammonia uptake by tea tree roots, promoted theanine synthesis, and favored the quality of tea leaves [59,60]. One of our research objectives was to link the species of microorganisms in the soil with the biochemical composition of tea. We found that some species of bacterial and fungal communities played a role in the soil by participating in nitrogen transformation, secreting enzymes, decomposing organic matter, and regulating soil pH, affecting tea biochemical composition [7,59]. Soil bacterial communities of *Bacillus* (Firmicutes) and *Rhodococcus* (Actinobacteria) have been reported to be used as plant growth-promoting rhizobacteria (PGPR) [61,62]. They can promote plant growth and facilitate the accumulation of phenolic metabolites in the plant [16], and there is a positive effect on the accumulation of biochemical components in tea. Similarly, a strong correlation was found between some of the soil flora and the biochemical composition of tea in our study. Fibrobacteres were strongly correlated with theanine in tea, and we hypothesized that because Fibrobacteres release ammonium nitrogen by decomposing soil organic matter, this would promote the uptake of nitrogen by the tea root system; increase the synthesis of glutamic acid, a precursor of theanine synthesis, in the tea root system; and elevate theanine content [63]. High-altitude soils may limit Fibrobacteres activity due to low temperatures, thereby reducing the theanine precursor supply. *Stachybotrys* acts as a saprophytic or weak pathogenic fungus. It may activate tea tree defense responses through its own metabolic activities, inducing the phenylpropane metabolic pathway and promoting catechin synthesis [64]. Lower levels of TP and TK in high-altitude soils may limit the metabolic activities of Fibrobacteres and Stachybotrys. This in turn reduces the regulation of tea tree metabolism. In our study, we found, interestingly, that there were several genera of fungi under the phylum Ascomycota which were significantly and positively correlated with the catechin content in tea (Figure 4). The interaction between soil microorganisms and the secondary metabolites of tea is still unclear, but we speculate that Ascomycota may release signaling molecules to influence precursors such as phenylalanine and increase catechin accumulation when decomposing plant cellulose or lignin [65,66]. The interactions between tea root microbial communities and tea secondary metabolism are complex. In the future, it is necessary to focus on the key signaling pathways of soil microorganisms regulating secondary metabolism and the directional influence of core metabolic pathways.

## 4. Materials and Methods

### 4.1. Study Sites and Sample Collection

The location of this study was the Fenghuang Mountain Tea Garden in Chaozhou City, Guangdong Province, China. It belongs to the Zhongshan terrain, with a mean annual temperature of 19 °C and a mean annual precipitation of 2119 mm. The selected tea plant was Oolong tea from Lingtou Dancong (*Camellia sinensis* cv. Lingtou Dancong). Consistent management of agronomic measures in tea plantations was conducted at different altitudes. Table 1 provides information on each sampling point. The tea trees have been cultivated at different altitudes for more than 50 years, using the same planting and cultivation management methods. Before taking soil samples, dead leaves and branches were removed from the soil surface. Then, the soil samples were taken from the root system of the tea tree at a depth of 5–20 cm. All soil samples were immediately placed on dry ice and transported to the laboratory. Soil samples were stored at 4 °C to determine soil enzyme activities and soil chemical properties, and then stored in a refrigerator at −80 °C for the subsequent analysis of soil microbial communities. Meanwhile, fresh leaves from a tea tree (one bud and two leaves) were collected from soil sampling sites in different altitudes, and the collected fresh leaves of the tea tree were quickly placed in dry ice and stored in a refrigerator at −80 °C for the subsequent determination of the biochemical constituents of tea leaves. Three biological replicates were carried out for different altitude groups.

### 4.2. Determination of Soil Physical and Chemical Properties

Soil pH (1:5 soil–water ratio) was measured using a digital pH meter. The soil organic matter (SOM) was determined using the K_2_Cr_2_O_7_ oxidation method [67]. Total nitrogen (TN) was determined using Kjeldahl digestion. Total phosphorus (TP) was extracted by the sodium hydroxide melting method and sodium bicarbonate extraction method, respectively. Total potassium (TK) was then determined using the anti-ammonia blue molybdenum colorimetric method, i.e., extracted using sodium hydroxide fusion and ammonium acetate extraction followed by flame photometry [68]. Catalase activity was measured by back-titrating residual H_2_O_2_ with KMnO_4_. Acid Phosphatase activity and Urease activity were determined using the disodium phosphate colorimetric method and phenol–sodium hypochlorite method, respectively [69].

### 4.3. Leaf Composition

Caffeine, theanine, and catechin concentrations were quantified using HPLC (Waters Alliance 26,952,489 UV/Vis; Waters Technologies, Milford, MA, USA). The tea samples stored in the refrigerator at −80 °C were dried and ground in a grinder to facilitate subsequent experiments to determine the content of inclusions in the tea leaves.

Determination of caffeine in tea samples [70]: 0.1 g of tea sample was mixed with 30 mL of 1.5% magnesium oxide solution in a water bath at 100 °C for 30 min. A total of 1 mL of the extract was filtered through a 0.22 μm Millipore membrane. Then, 10 μL of the filtrate was injected into an XSelect HSS C18 SB column at a flow rate of 0.9 mL/min and a column temperature of 35 ± 1 °C (4.6 × 250 mm, 5 μm). The mobile phase comprised 100% methanol (A) and 100% ultrapure water (B). Caffeine was detected at 280 nm.

Determination of theanine in tea samples [70]: 0.1 g of tea sample was added to 10 mL of boiling ultrapure water and then extracted in a constant temperature water bath at 100 °C for 30 min. The supernatant was filtered using a 0.22 μm Millipore membrane, and then 10 μL of filtrate was injected onto an RP-C18 column (4.0 × 250 mm, 5 um) at a flow rate of 0.5 mL/min and a column temperature of 35 ± 1 °C. The theanine content was detected at a wavelength of 210 nm.

Determination of catechins in tea samples [71]: 0.2 g of the tea sample was extracted by adding 8 mL of 70% methanol, and then 1 mL of the supernatant was filtered using a 0.22 mm Millipore membrane. The filtrate was injected onto an XSelect HSS C18 SB column (4.6 × 250 mm, 5 μm), with catechin monomers in a gradient elution using a 0.1% aqueous formic acid solution (A) and 100% acetonitrile (B) as the mobile phase. The gradient elution program was used. The catechins were detected at 280 nm.

### 4.4. DNA Extraction and Illumina Sequencing

Total DNA was extracted from 0.25 g of the soil samples using the Power Soil DNA Isolation kit (Mo Bio Laboratories Inc., Carlsbad, CA, USA). After extracting genomic DNA from the samples, we amplified the V3–V4 regions of the bacterial 16S rRNA gene with the primer set of 341F (5′-CCTACGGGNGGCWGCAG-3′) and 805R (5′-GGACTACHVGGGTATCTAAT-3′) [72], and the ITS region of the fungi with the primer set of ITS (ITS3_KYO2: GATGAAGAACGYAGYRAA; ITS4: TCCTCCGCTTATTGATATGC). They were sequenced on an Illumina MiSeq platform (Gene Denovo Biotechnology Co., Ltd., Guangzhou, China).

### 4.5. Statistical Analysis

The mean and standard error of three replicates were calculated for internal components, soil chemical properties, and enzyme activities in tea samples using Microsoft Excel 2021. The significant differences between the samples were tested using one-way analysis of variance (ANOVA) in SPSS 27 (SPSS Inc., Chicago, IL, USA) and Duncan’s multiple range test. Graphing tea leaf inclusions and soil chemical properties was conducted with Graphpad Prism 9.5.0 (GraphPad Software, Inc., La Jolla, CA, USA). Microbiome informatics analysis was performed on the Omicsmart platform (https://www.omicsmart.com; accessed on 20 May 2025). Analyzing the β-diversity of bacteria and fungi in soil was performed using non-metric multidimensional scaling (NMDS) based on the Bray–Curtis distance algorithm. We used redundancy analysis (RDA) to analyze the relationship between soil chemical properties and bacterial and fungal communities in soils. Pearson correlation analysis was used for the correlation between the relative abundance of soil microorganisms and tea inclusions.

## 5. Conclusions

Our results revealed that changes in altitude resulted in significant changes in the pH, soil chemical properties, and soil enzyme activities of tea garden soils. However, the direct effect of the elevation on microbial communities may have been masked by the indirect effect of environmental factors. This resulted in insignificant differences in the spatial composition of microbial communities in the rhizosphere soil. Soil fungal communities were most abundant at low altitudes under the synergistic effect of ecotope differentiation strategies driven by environmental factors and nutrient limitations. Meanwhile, our results suggest that pH and TP in soil were the driving factors influencing soil bacterial community composition along the altitude gradient. Tea content at low altitudes is mainly regulated by soil chemical properties, and the quality of tea is at its highest. Meanwhile, we found a strong relationship between microbial communities and tea quality. Fungal diversity can be a factor in measuring tea quality indicators. We also found a strong correlation between the key functional group of bacteria, Fibrobacteres, and tea quality components. Our study shows that changes in altitude cause changes in soil chemical properties. This alters the ecological adaptation strategies of some microorganisms in the soil, while affecting tea quality components. In this study, by linking soil chemical properties and microorganisms with the main internal components of tea, we provide a new theoretical perspective to study the relationship between tea quality and soil.

## Figures and Tables

**Table 1:** Correlation analysis between the soil chemical properties and the relative abundance of a dominant phylum or genus. Values are correlation coefficients, and bold numbers indicate a significant correlation (* *p* < 0.05, ** *p* < 0.01).

Phylum/Genus	pH	SOM	TN	TP	TK	Catalase	Acid Phosphatase	Urease
**Bacteria**								
Patescibacteria	0.38	−0.43	−0.52	−0.24	−0.10	0.17	0.02	0.76 *
Cyanobacteria	−0.29	0.55	−0.02	0.12	0.02	−0.36	0.81 *	−0.14
Fibrobacteres	−0.71 *	0.24	0.05	0.90 **	0.86 **	−0.55	−0.26	−0.24
Nitrospirae	−0.74 *	0.71 *	0.33	0.64	0.29	−0.50	0.52	−0.79 *
Rhodococcus	−0.60	0.07	−0.17	0.83 *	0.81 *	−0.50	−0.21	−0.02
**Fungi**								
Mortierellomycota	−0.73 *	0.47	0.00	0.85 **	0.55	−0.58	0.17	−0.57
Humicola	−0.68 *	0.48	0.28	0.54	0.30	−0.68 *	0.23	−0.22
Cephaliophora	0.73 *	−0.55	−0.18	−0.36	−0.27	0.53	−0.18	0.37
Mortierella	−0.73 *	0.47	0.00	0.85**	0.55	−0.58	0.17	−0.57
Stachybotrys	−0.80 **	0.57	0.23	0.89 **	0.48	−0.68*	0.27	−0.50
Pseudogymnoascus	−0.70 *	0.35	0.12	0.41	0.33	−0.57	−0.02	−0.27

**Table 2:** Correlation analysis between soil microbial diversity and green tea’s major composition or quality indices. Values are correlation coefficients, and bold numbers indicate a significant correlation (** *p* < 0.01).

Microbial Diversity Index	Theanine	Caffeine	Catechin	Theanine/Caffeine	Theanine/Catechin
Bacterial richness	0.33	0.15	0.02	0.37	0.33
Fungal Shannon diversity	−0.27	−0.05	0.20	−0.33	−0.27
Fungal richness	0.80 **	0.40	0.13	0.85 **	0.80 **
Fungal Shannon diversity	−0.35	−0.58	−0.23	−0.27	−0.36

**Table 3:** Latitude, longitude, and elevation of each sampling point.

	Low Altitude	Mid-Altitude	High Altitude
Latitude	116°44′48″ N	116°41′54″ N	116°41′7″ N
Longitude	23°55′7″ E	23°55′44″ E	23°56′35″ E
Altitude (m)	396.59	517.97	623.44 m

## Data Availability

The original contributions presented in this study are included in the article. Further inquiries can be directed to the corresponding authors.

## Supplementary Materials

The following supporting information can be downloaded at: https://www.mdpi.com/article/10.3390/plants14111642/s1, Table S1: Soil chemical properties at three different altitudes; Figure S1: The relative abundance of the bacterial and fungal community at the genus level; Table S2: Correlation analysis between soil microbial diversity and soil chemical properties; Table S3: Correlation analysis between soil microbial diversity and Soil chemical properties; Figure S2: Composition of catechins in tea leaves.
