# Supplementary material for: Effects of Altitude on Tea Composition: Dual Regulation by Soil Physicochemical Properties and Microbial Communities
Source: Plants (Basel). 2025 May 28;14(11):1642. doi: 10.3390/plants14111642 (PMC12157788; doi:10.3390/plants14111642)
Supplement: Supplementary file 1 [file plants-14-01642-s001.zip › plants-3591306-supplementary.pdf]

| Soil properties | L-A | M-A | H-A |
|---|---|---|---|
| pH | 4.36±0.14[ab] | 4.05±0.04[b] | 4.53±0.05[a] |
| SOM（g/kg） | 21.23±3.40[b] | 33.87±3.82[a] | 25.13±0.75[ab] |
| TN（g/kg） | 1.06±0.12[a] | 1.12±0.09[a] | 1.22±0.02[a] |
| TP（mg/kg） | 0.73±0.19[a] | 0.61±0.05[a] | 0.20±0.18[b] |
| TK（mg/kg） | 12.27±1.39[a] | 8.05±0.62[b] | 5.98±0.40[b] |
| Catalase(ml/g) | 2.99±0.27[ab] | 2.05±0.37[b] | 3.44±0.27[a] |
| Acid Phosphatase(mg/kg) | 384.40±33.45[c] | 812.39±17.43[a] | 608.61±57.52[b] |
| Urease(mg/kg) | 535.58±129.93[a] | 301.27±67.04[a] | 420.31±54.33[a] |

**Table S1:** Soil chemical properties at three different altitudes. L-A, Low Altitude, M-A, Middle Altitude, H-A, High Altitude. (mean ± standard error, n=4).

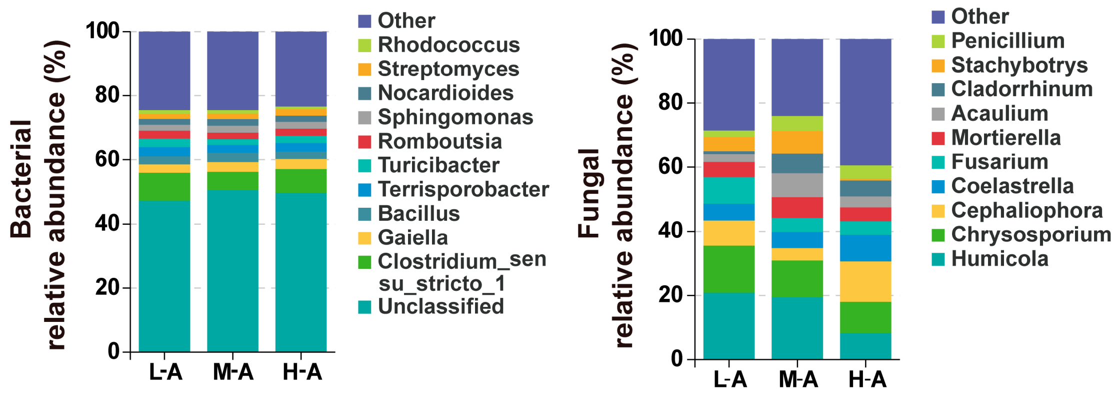


**Figure S1:** The relative abundance of the bacterial and fungal community at the genus level (n=3). L-A, Low Altitude, M-A, Middle Altitude, H-A, High Altitude.

| | pH | SOM | TN | TP | TK | Catalase | Acid Phosphatase | Urease |
|---|---|---|---|---|---|---|---|---|
| **Theanine** | -0.12 | -0.38 | -0.47 | 0.64 | **0.92**** | -0.11 | -0.64 | 0.32 |
| **Caffeine** | -0.44 | -0.05 | -0.64 | **0.86**** | **0.79*** | -0.43 | -0.12 | 0.21 |
| **Catechin** | **-0.73*** | 0.36 | -0.31 | **0.96**** | 0.53 | **-0.68*** | 0.16 | -0.13 |
| **Theanine/ Caffeine** | -0.04 | -0.42 | -0.33 | 0.52 | **0.86**** | -0.02 | **-0.74*** | 0.28 |
| **Theanine/ Catechin** | 0.01 | -0.48 | -0.47 | 0.54 | **0.92**** | -0.01 | **-0.73*** | 0.37 |

**Table S2:** Correlation analysis of major components in tea with soil chemical properties. Bold indicates a strong correlation.

| Microbial diversity index | pH | SOM | TN | TP | TK | Catalase | Acid Phosphatase | Urease |
|---|---|---|---|---|---|---|---|---|
| **Bacterial richness** | -0.23 | -0.15 | -0.28 | 0.03 | 0.35 | -0.30 | -0.37 | -0.03 |
| **Fungal Shannon diversity** | -0.15 | 0.17 | 0.13 | 0.22 | -0.23 | -0.20 | 0.43 | 0.12 |
| **Fungal richness** | 0.07 | -0.38 | -0.15 | 0.34 | **0.73*** | -0.07 | -0.65 | 0.23 |
| **Fungal Shannon diversity** | -0.17 | 0.23 | 0.33 | -0.29 | -0.45 | 0.00 | 0.10 | -0.53 |

**Table S3:** Correlation analysis between soil microbial diversity and Soil chemical properties.Bold indicates a strong correlation.

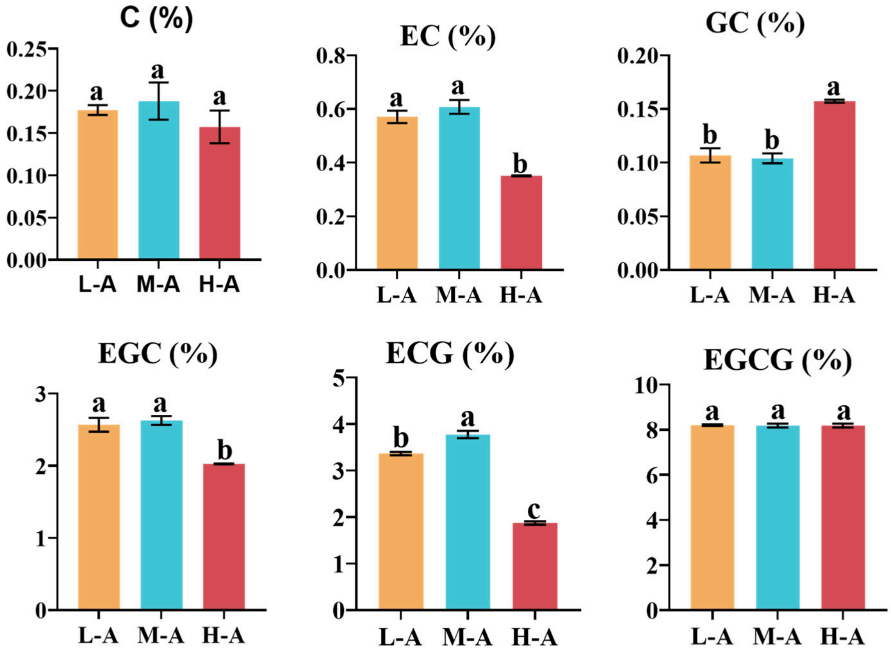


**Figure S2:** Composition of catechins in tea leaves. The data represent means ± standard error (n=3). Letters denote statistically significant differences between seasons ($p < 0.05$).C; catechin, EC; epicatechin, GC; gallocatechin, EGC; epigallocatechin, ECG, epicatechin-3-gallate, EGCG; epigallocatechin gallate.
